# DELLA proteins interact with HAC1 and control plant growth via transcriptional regulation of the growth repressor gene *ERF2*

**DOI:** 10.1093/plcell/koag235

**Published:** 2026-08-10

**Authors:** Hiroki Ando, Akira Nozawa, Hidetaka Kosako, Tatsuya Sawasaki, Yohsuke Takahashi, Jutarou Fukazawa

**Affiliations:** Program of Basic Biology, Graduate School of Integrated Science for Life, Hiroshima University, Higashi-Hiroshima 739-8526, Japan; Division of Cell-Free Sciences, Proteo-Science Center, Premier Institute for Advanced Studies, Ehime University, 3 Bunkyo-cho, Matsuyama, Ehime 790-8577, Japan; Division of Cell Signaling, Institute of Advanced Medical Sciences, Tokushima University, Tokushima 770-8503, Japan; Division of Cell-Free Sciences, Proteo-Science Center, Premier Institute for Advanced Studies, Ehime University, 3 Bunkyo-cho, Matsuyama, Ehime 790-8577, Japan; Program of Basic Biology, Graduate School of Integrated Science for Life, Hiroshima University, Higashi-Hiroshima 739-8526, Japan; Program of Basic Biology, Graduate School of Integrated Science for Life, Hiroshima University, Higashi-Hiroshima 739-8526, Japan

## Abstract

DELLA proteins are negative regulators of gibberellin (GA) signaling. Under GA-deficient conditions, DELLA accumulates in the nucleus and interacts with numerous transcription factors (TFs) via the GRAS domain. Recent studies have shown that DELLA interacts with TFs via the LHR1 subdomain and histone H2A via the PFYRE subdomain, respectively. It is essential for DELLA-mediated global transcription reprograming. DELLA exhibits strong transcriptional activity and acts as a co-activator of GAI-ASSOCIATED FACTOR1 (GAF1)/IDD2. The N-terminus of DELLA is necessary for strong transcriptional activity, but the regulatory activities and mechanisms of DELLA remain unclear. Therefore, we screened DELLA-interacting factors using the proximity biotinylation enzyme AirID and identified HISTONE ACETYLTRANSFERASE OF THE CBP FAMILY1 (HAC1). HAC1-mediated acetylation of histone H3 at the GAF1 target chromatin was necessary for transcriptional activation by the GAF1–DELLA complex. Additionally, the HAC1-mediated transcriptional activity of DELLA was involved in regulating plant growth. Moreover, *ETHYLENE RESPONSE FACTOR2* (*ERF2*) was identified as a growth repressor gene regulated by the GAF1–DELLA–HAC1 complex. Here, we showed that DELLA proteins regulate plant growth via HAC1-mediated transcriptional regulation of *ERF2*.

## Introduction

Gibberellins (GA) are plant hormones that promote germination, growth, and flowering. The DELLA protein acts as a negative regulator in the GA signaling pathway. GA-deficient or DELLA-accumulated mutants exhibit low germination rates, dwarf, and delayed flowering phenotypes (Sun 2008; Bao et al. 2020). In GA-sufficient conditions, DELLA interacts with a GA receptor, GIBBERELLIN-INSENSITIVE DWARF1 (GID1) (Ueguchi-Tanaka et al. 2005; Griffiths et al. 2006). Notably, DELLA proteins are recognized by an SCF complex including the SLEEPY1 F-box protein (SCF^SLY1/GID2^) (McGinnis et al. 2003; Sasaki et al. 2003) and degraded via the 26S proteasome pathway. Presently, 5 DELLA proteins have been identified in *Arabidopsis thaliana*: REPRESSOR of *ga1-3* (RGA), GA-INSENSITIVE (GAI), RGA-LIKE1 (RGL1), RGL2, and RGL3. DELLA proteins belong to the GRAS family and are plant-specific transcriptional regulators. DELLA proteins possess DELLA and TVHYNP motifs in the N-terminal region, which are necessary for their interaction with GID1 (Dill et al. 2001; Murase et al. 2008), and a GRAS domain in the C-terminal region that confers transcriptional regulatory function and is conserved among all GRAS family proteins. Although DELLA lacks an apparent DNA-binding motif, ChIP or ChIP-seq analyses have shown that DELLA is associated with target promoters (Zentella et al. 2007). Further studies indicate that DELLA proteins interact with numerous TFs via the GRAS domain (la Rosa et al. 2014; Lantzouni et al. 2020). Previously, the functions of DELLA have been explained by 2 models. The titration model proposes that DELLA binds to and inhibits the DNA-binding ability of TFs such as PHYTOCHROME INTERACTING FACTORs (PIFs) (de Lucas et al. 2008; Feng et al. 2008). The co-activator model proposes that DELLA functions as a co-activator for TFs such as the INDETERMINATE DOMAIN (IDD) family proteins (Fukazawa et al. 2014; Yoshida et al. 2014). Recently, Huang et al. (2023) demonstrated that DELLA primarily acts on chromatin, highlighting the importance of detailed analysis of its chromatin-associated functions. DELLA protein interacts with many TFs via the LHR1 subdomain and with histone H2A via the PFYRE subdomain, and the DELLA-H2A interaction is essential for DELLA-mediated global transcription reprograming. In contrast, the function of the N-terminal region of DELLA remains largely unknown, aside from its role in GA-dependent degradation through GID1 binding via the DELLA motif. Analysis of SLENDER RICE1 (SLR1), a DELLA protein in *Oryza sativa*, revealed the presence of a transcriptional activation domain in its N-terminal region (Hirano et al. 2012). Similarly, analyses of GAI, a DELLA protein in *Arabidopsis*, have shown that the N-terminal region plays a key role in transcriptional activation (Figure S1). The transactivation domain (TAD) of transcriptional activators contains ξ–φ–x–x–φ–φ, φ–x–x–φ–φ, or φ–φ–x–x–φ motifs (acidic residue [ξ]; bulky hydrophobic residue [φ]) (Lee et al. 2009; Denis et al. 2012). The N-terminal region of DELLA proteins also contains TAD. A previous study reported that DELLA proteins recruit the mediator complex subunit MED15 to co-activate transcription (Hernández-García et al. 2024). Collectively, elucidating the chromatin-associated functions of DELLA remains an important issue, and the mechanisms underlying strong DELLA-mediated transcriptional activation are not yet fully understood.

Previously, we identified a DELLA-interacting factor, GAI-ASSOCIATED FACTOR1 (GAF1)/IDD2 (Fukazawa et al. 2014). Additionally, GAF1 interacts with TOPLESS/TOPLESS-RELATED (TPL/TPR) to repress the transcription of target genes (Fukazawa et al. 2014). DELLAs and TPR are co-activators and co-repressor of GAF1, respectively (Fukazawa et al. 2014). Research indicates that DELLA is degraded in the presence of GA (McGinnis et al. 2003; Griffiths et al. 2006). GAs convert the GAF1 complex from a transcriptional activator to a repressor. Additionally, the GAF1 complex is involved in GA feedback regulation (Fukazawa et al. 2017). TPL/TPR recruits histone deacetylases (HDACs) and represses transcription through deacetylation of the target chromatin (Krogan et al. 2012; Yusuf et al. 2025). In *Nicotiana tabacum*, the histone H3 of *NtGA20ox1*, which is subject to GA feedback regulation, is acetylated in the absence of GA (Fukazawa et al. 2010). Collectively, these findings suggest that DELLA activates transcription through histone modifications; however, the underlying molecular mechanisms remain unclear.

Histone acetylation is reversible and dynamically regulated by histone acetyltransferases (HATs) and HDACs (Chen et al. 2024). HAT is a well-known transcriptional co-activator that modulates the chromatin structure. CREB-binding protein (CBP)/p300 is a subfamily of HAT that acetylates lysine residues of histones H3 and H4 (Earley et al. 2007), thereby leading to chromatin relaxation and transcriptional activation. HISTONE ACETYLTRANSFERASE OF THE CBP FAMILY1 (HAC1), HAC2, HAC4, HAC5, and HAC12 are orthologs of CBP/p300 in *Arabidopsis* (Bordoli et al. 2001). Analysis of *hac* mutants indicates that HAC1 acts as a major HAT among HAC family proteins (Li et al. 2014). For example, HAC1 has been reported to play a key role in the transcriptional activation of MYC2, the master TF in jasmonic acid (JA) signaling (An et al. 2017), and as a transcriptional activator of the TGACG-BINDING FACTORs (TGAs)-NONEXPRESSOR OF PATHOGENESIS-RELATED GENES1 (NPR1) complex in salicylic acid (SA) signaling (Jin et al. 2018; Yun et al. 2024). Overall, these studies highlight the importance of transcriptional activation by TFs and HAC1 in plant hormone signaling.

Traditionally, the yeast two-hybrid (Y2H) assay has been used to screen for interacting proteins. Considering Y2H screening depends on transcriptional activation, it is difficult to employ factors with transcriptional activation capacity as bait proteins. Since the N-terminal region of DELLA proteins exhibits strong transcriptional activity in yeast, the screening of DELLA-interacting proteins was performed using the C-terminal region as the bait protein. Although many TFs that interact with the C-terminal region of DELLA have been identified (la Rosa et al. 2014; Lantzouni et al. 2020), little is known regarding factors that interact with the N-terminal region. In this study, we comprehensively screened factors that interact with full-length DELLA using the proximity biotinylation enzyme AirID (Kido et al. 2020), independent of transcriptional activation. We identified HAC1 as a novel interacting factor of DELLA, and HAC1 interacts with the N-terminal region of DELLA, which is crucial for its transcriptional activation. HAC1 promotes histone H3 acetylation and the expression of DELLA target genes. Moreover, *ETHYLENE RESPONSE FACTOR2* (*ERF2*), a growth repressor gene, was identified as a novel DELLA target gene. We concluded that DELLA proteins regulate plant growth via HAC1-mediated transcriptional regulation of the growth repressor gene *ERF2*. Here, we aimed to elucidate the chromatin-associated mechanism underlying DELLA-mediated transcriptional activation and identified HAC1 as a chromatin co-activator acting on GAF1 target genes.

## Results

### Screening of DELLA-interacting proteins using AirID

To identify novel factors involved in the transcriptional activity of DELLA, we screened for DELLA-interacting proteins using the proximity biotinylation enzyme AirID (Kido et al. 2020). AirID-fused DELLA proteins were transiently expressed in T87 cultured cells. Immunoblotting using an anti-AGIA antibody confirmed the expression of AGIA-tagged AirID fused to GAI and RGA (Figure S2a). In this system, DELLA-interacting proteins were effectively biotinylated by AirID-GAI or AirID-RGA proteins. To confirm the presence of biotinylated proteins in T87 cultured cells expressing AirID-GAI or AirID-RGA, we performed immunoblotting using an anti-biotin antibody. Notably, we observed bands corresponding to proteins that were presumed to be biotinylated in cells expressing AGIA-AirID-GAI or AGIA-AirID-RGA (Figure S2b). To investigate the effect of the fused AirID protein on the transcriptional activity of DELLA, we compared the transcriptional activity of DELLA and AirID-fused DELLA proteins in a transactivation assay. Both GAF1-GAI and GAF1-AirID-GAI activated the *GA20ox2* promoter, which is one of the target genes of the GAF1–DELLA complex (Figure S3). Therefore, it was inferred that the fusion with AirID did not inhibit the transcriptional activity of DELLA. Mass spectrometric analysis of biotinylated peptides from T87 cultured cells transiently expressing AGIA-AirID-DELLA identified several candidate proteins that potentially interact with DELLA (Table S1). Specifically, these candidates include known DELLA-interacting proteins such as GID1A (Griffiths et al. 2006; Nakajima et al. 2006), GAF1, GAF2 (Fukazawa et al. 2014), PIF3 (de Lucas et al. 2008; Feng et al. 2008), AUXIN RESPONSE FACTOR7 (ARF7) (Oh et al. 2014), and MEDIATOR15 (MED15) (Hernández-García et al. 2024; Ohama et al. 2025). Overall, these results indicate that using the AirID screening technique is effective in identifying DELLA-interacting proteins.

### DELLA directly interacts with HAC1 via the N-terminal region in the nucleus

**Figure 1:**
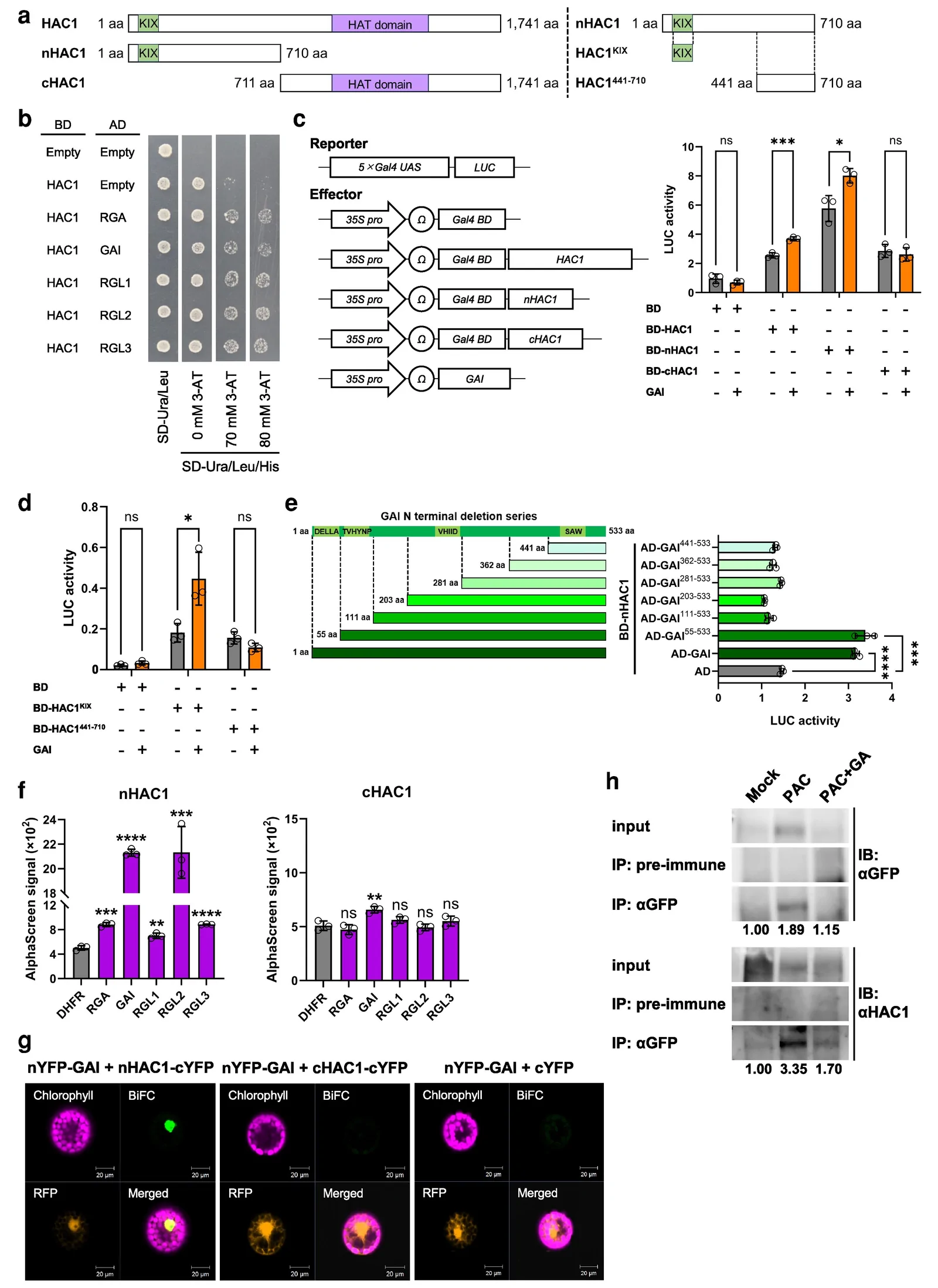
N-terminal of GAI directly interacts with HAC1 in the nucleus. a) Schematic diagrams depicting the structure of HAC1. b) Yeast two-hybrid assay shows the interaction of AtDELLAs with HAC1. The bait protein was fused to the Gal4 DNA-binding domain (BD), while the prey protein was fused to the Gal4 transactivation domain (AD). Transformed yeast cells were grown on SD-Ura/Leu medium and SD-Ura/Leu/His medium supplemented with 0-80 mM 3-amino-1,2,4-triazole (3-AT). c) Plant two-hybrid assay showing interaction of GAI with HAC1 and nHAC1 in the mesophyll protoplasts. Each effector was introduced into protoplasts derived from Col-0 mesophyll cells with the reporter, and LUC/rLUC activity was measured after 20 h of culture. d, e) Plant two-hybrid assay was performed to identify the GAI-interacting region in nHAC1 (d) and the nHAC1-interacting region in GAI (e). f) AlphaScreen assay showing the direct interaction between AtDELLAs and nHAC1 or cHAC1 in vitro. Dihydrofolate reductase (DHFR) was used as a negative control. g) Bimolecular fluorescence complementation (BiFC) assay showed the interaction between GAI and nHAC1 in the nucleus in Col-0 mesophyll protoplasts. The co-expression of nYFP-GAI and cYFP empty vector served as a negative control. Scale bars = 20 μm. h) Co-IP assay showing that HAC1 was co-immunoprecipitated by GFP-RGA. 7-d-old *RGApro:GFP-RGA* transgenic seedlings were treated with or without 1 μM PAC or 10 μM GA_3_ for 7 d. GFP-RGA was immunoprecipitated using anti-GFP antibodies and immunoblotted using anti-GFP and anti-HAC1 antibodies. The value of Mock was set as 1.00. Error bars indicate standard deviation (SD) of 3 biological replicates (*n* = 3). Asterisks indicate significant differences by Student's *t-test* (ns; not significant, *P < 0.05, **P < 0.01, ***P < 0.001, ****P < 0.0001).

Among the candidate DELLA-interacting proteins, we focused on HAC1 because histone acetylation is closely associated with transcriptional activation. Histone acetylation maintains a relaxed chromatin structure and promotes the transcription of target genes. HAC1 has a KIX domain at the N-terminal region that mediates interaction with TFs (Kumar et al. 2018; Hernández-García et al. 2024), and a HAT domain at the C-terminal region (Thakur et al. 2013) (Fig. 1a). The KIX domain interacts with the TAD of TFs (L. W. Lee and Mapp 2010), and the TAD exists in the N-terminal region of DELLAs (Hernández-García et al. 2019, 2024). To investigate the interaction between DELLA and HAC1, we performed Y2H and plant two-hybrid assays using protoplasts derived from mesophyll cells. Notably, the Y2H assay demonstrated that full-length HAC1 interacted with all AtDELLA (Fig. 1b, Figure S4a). Additionally, Y2H showed that HAC1^1–710aa^ (nHAC1) interacted with all AtDELLAs, whereas HAC1^711–1741aa^ (cHAC1) did not interact with any AtDELLA (Figure S4b, c). Next, we investigated the interaction between HAC1 and GAI in plant cells using the plant two-hybrid assay in protoplasts derived from mesophyll cells. The full-length HAC1 and nHAC1 interacted with GAI, but cHAC1 did not interact with GAI (Fig. 1c). The plant two-hybrid results are compatible with, rather than directly confirming, the physical interaction observed in yeast. These results are consistent with the Y2H results. To investigate the region of interaction between HAC1 and GAI, nHAC1 was further subdivided into subdomains. The KIX domain of HAC1 interacted with GAI, whereas the 441 to 710 aa region of HAC1 did not interact with GAI (Fig. 1d). We further examined whether HAC1*Δ*KIX and nHAC1*Δ*KIX, lacking the KIX domain, mediated the interaction with AtDELLAs. Unexpectedly, HAC1*Δ*KIX and nHAC1*Δ*KIX were suggested to interact with all AtDELLAs (Figure S4d–f). To identify the interaction region of GAI with nHAC1, a plant two-hybrid assay was performed following the deletion of a specific region in GAI. nHAC1 interacted with full-length GAI and the 55 to 533 aa region (deleted DELLA motif of GAI), but did not interact with GAI lacking the 1 to 110 aa region, including the TVHYNP motif (Fig. 1e). Collectively, these results indicate that GAI interacts with HAC1 via the 55 to 110 aa region. The AlphaScreen assay was performed to investigate in vitro protein–protein interactions using recombinant biotin-ligation site (bls)-tagged nHAC1, nHAC1*Δ*KIX, and cHAC1 (nHAC1-, nHAC1*Δ*KIX-bls, bls-cHAC1), and FLAG-tagged AtDELLAs (RGA-, GAI-, RGL1-, RGL2-, RGL3-FLAG) proteins (Figure S5a). The AlphaScreen assay is an in vitro proximity-based assay in which donor and acceptor beads generate a chemiluminescent signal upon interaction, and it was used to detect protein–protein interactions. A significant fluorescence signal was detected due to the interaction between nHAC1 and AtDELLAs, with particularly strong signals for the interaction between nHAC1 and GAI or RGL2 (Fig. 1f). In contrast, the interactions between nHAC1*Δ*KIX and each of the DELLA proteins were weak (Figure S5b). Additionally, cHAC1 did not interact with DELLA proteins except GAI, with only a weak interaction observed between cHAC1 and GAI (Fig. 1f). These results suggest that the KIX domain of HAC1 is critical for the interaction and may even be sufficient for interaction with DELLA. Additionally, bimolecular fluorescence complementation (BiFC) analysis was performed on Col-0 mesophyll protoplasts to further investigate the protein–protein interactions between GAI and nHAC1 in plant cells. Although no YELLOW FLUORESCENT PROTEIN (YFP) signal was observed following nYFP-GAI co-transfection with cYFP and cHAC1-cYFP, the reconstructed YFP signal, caused by the interaction between nYFP-GAI and nHAC1-cYFP, was observed in the nuclei of mesophyll protoplasts (Fig. 1g). To confirm the interaction between nHAC1 and DELLA in vivo, we performed co-immunoprecipitation (co-IP) assay using the T87 cultured cells within HA-tagged nHAC1, nHAC1*Δ*KIX, or cHAC1 and FLAG-tagged GAI were expressed. HA-nHAC1 and HA-nHAC1*Δ*KIX were co-immunoprecipitated with FLAG-GAI, but HA-cHAC1 was not (Figure S6). Furthermore, we conducted a co-IP assay using *RGApro:GFP-RGA* transgenic plants. Notably, the GFP-RGA protein interacts with endogenous HAC1 protein under GA-deficient conditions induced by treatment with paclobutrazol (PAC), a GA biosynthesis inhibitor (Fig. 1h). Overall, these results suggest that DELLA interacts with HAC1 in the nucleus via the N-terminal region.

### Loss of HAC1 attenuates DELLA-mediated growth inhibition and reduces DELLA-dependent transcriptional activation

**Figure 2:**
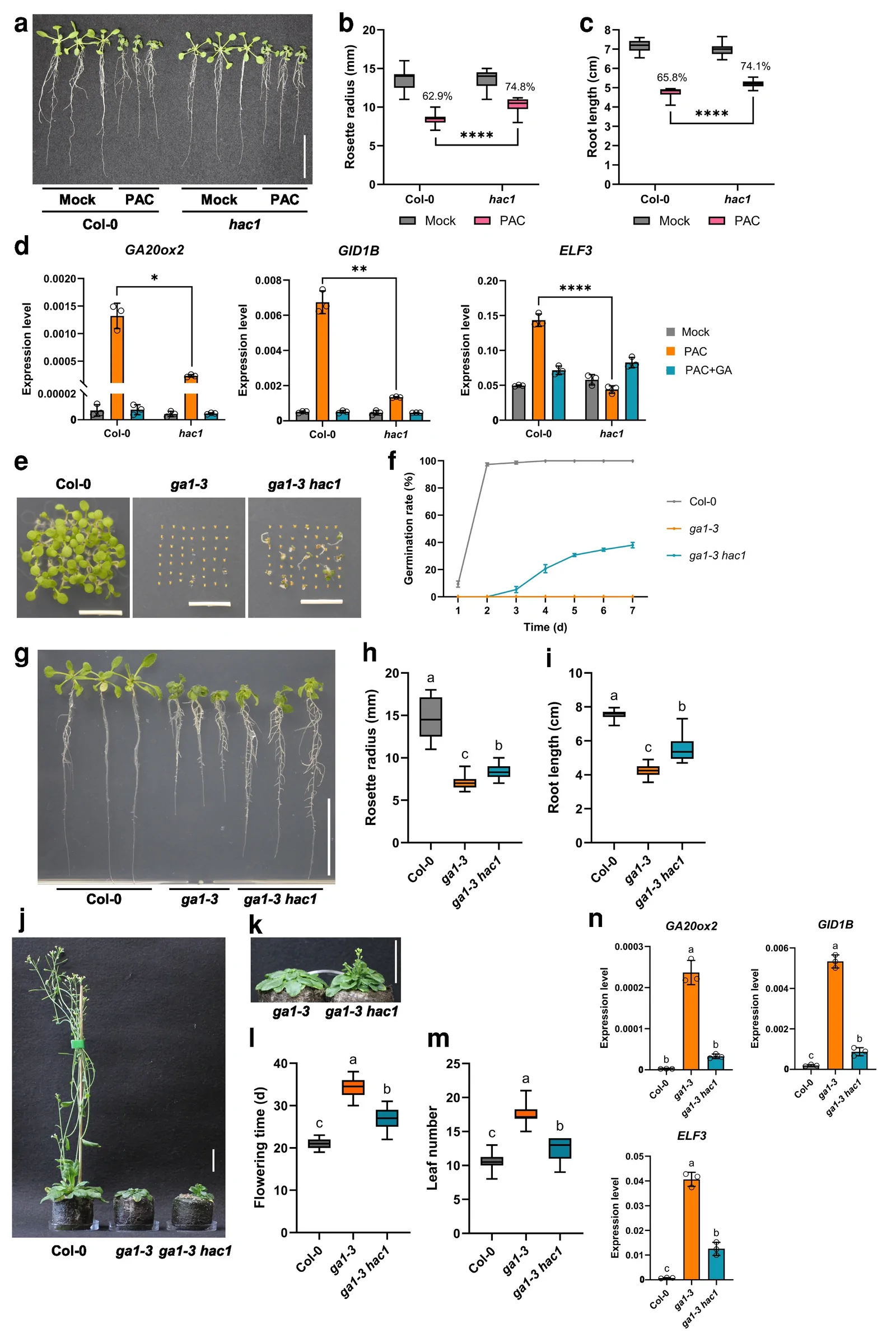
GA deficiency-induced growth inhibition and upregulation of GAF1-target genes were attenuated by loss of HAC1. a) Phenotypes of Col-0 and *hac1* seedlings grown on half-strength MS agar medium. 7-d-old Col-0 and *hac1* seedlings were treated with or without 1 μM PAC for 7 d. Scale bar = 3 cm. b, c) Rosette radius (b) and root length (c) of 7-d-old Col-0 and *hac1* seedlings were treated with or without 1 μM PAC for 7 d; *n* = 13 biologically independent samples. The numbers above the bars indicate the percentage of PAC/Mock. d) Expression levels of GAF1-targeted genes in Col-0 and *hac1* seedlings. 7-d-old Col-0 and *hac1* seedlings were treated with or without 1 μM PAC or 10 μM GA_3_ for 7 d. The expression levels of the target genes were normalized to that of *UBQ11*. Error bars indicate SD of 3 biological replicates (*n* = 3). e) Seeds of Col-0, *ga1-3*, and *ga1-3 hac1* were germinated on half-strength MS agar medium, and after 7 d a representative picture of each plant was taken. Scale bar = 1 cm. f) Germination rate of Col-0, *ga1-3*, and *ga1-3 hac1* seeds at 1 to 7 d after stratification on half-strength MS agar medium. g) Phenotypes of Col-0, *ga1-3*, and *ga1-3 hac1* seedlings grown on half-strength MS agar medium for 14 d. Scale bar = 3 cm. h, i) Rosette radius (h) and root length (i) of Col-0, *ga1-3*, and *ga1-3 hac1* seedlings grown on half-strength MS agar medium for 14 d; n ≥ 17 biologically independent samples. j, k) Phenotypes of 45-d-old (j) and 55-d-old (k) Col-0, *ga1-3*, and *ga1-3 hac1* double mutant plants. Scale bar = 3 cm. l, m) Flowering time (l) and leaf number (m) of Col-0, *ga1-3*, and *ga1-3 hac1* seedlings grown under long-day conditions; *n* ≥ 15 biologically independent samples. n) Expression levels of GAF1-target genes in Col-0, *ga1-3*, and *ga1-3 hac1* seedlings grown on half-strength MS agar medium for 14 d. The expression levels of the target genes were normalized to that of *UBQ11*. Error bars indicate the SD of 3 biological replicates (*n* = 3). In all boxplots shown in (b, c), (h, i), and (l, m), center lines and box edges are medians and the interquartile range (IQR), respectively. Whiskers extend to the lowest and highest data points within 1.5 times the IQR below and above the lower and upper quartiles, respectively. Asterisks indicate significant differences by Student's *t* test (*P < 0.05, **P < 0.01, ****P < 0.0001). Different letters above the bars indicate significant differences (one-way ANOVA: Tukey–Kramer's multiple comparisons test, *P* < 0.05).

DELLA directly interacts with HAC1 in the nucleus, suggesting that HAC1 may contribute to DELLA-mediated transcriptional activation and growth regulation. To test this hypothesis, we examined the phenotypes of *hac1* mutant under GA-deficient conditions. PAC treatment inhibited growth and root elongation in Col-0 plants; however, this growth inhibition was partially restored in the *hac1* mutant (Fig. 2a–c), thereby indicating that HAC1 contributed to growth inhibition under GA-deficient conditions. In previous studies, we reported that the GAF1–DELLA complex activates GAF1 target genes, including *GA20ox2*, *GID1B*, and *ELF3*, under GA-deficient conditions that cause DELLA accumulation (Fukazawa et al. 2014, 2017, 2021). Therefore, we compared the expression levels of GAF1 target genes in Col-0 and the *hac1* mutant to investigate the contribution of HAC1 to the activation of GAF1 target genes under GA-deficient conditions. The results of the expression level analysis showed that *GA20ox2*, *GID1B*, and *ELF3* expression levels significantly increased in Col-0 treated with PAC. In contrast, the increase in expression levels of these genes under GA-deficient conditions was attenuated in the *hac1* mutant (Fig. 2d).

*ga1-3*, a GA-deficient mutant, exhibited severe growth defects, including low germination rates, growth inhibition, and late-flowering. To investigate the role of HAC1-mediated DELLA transcriptional activation in plant growth regulation, we generated a *ga1-3 hac1* double mutant. Notably, the germination rates of the Col-0, *ga1-3*, and *ga1-3 hac1* double mutant were 100%, 0%, and 40%, respectively (Fig. 2e, f). Compared with *ga1-3*, the *ga1-3 hac1* double mutant exhibited improved rosette radius and root length (Fig. 2g–i). Additionally, the late-flowering phenotype of *ga1-3* was restored in the *ga1-3 hac1* double mutant (Fig. 2j–m). Collectively, these results indicate that HAC1 is required for DELLAs to regulate plant growth inhibition at multiple stages.

Since *ga1-3* is a GA biosynthesis mutant that does not fully mimic DELLA accumulation states, similar experiments were conducted using the gain-of-function mutant *gai-1*. Notably, *gai-1* exhibits a dwarf phenotype, but the *gai-1 hac1* double mutant demonstrated a partially restored phenotype (Figure S7).

To investigate the role of HAC1 in the DELLA-dependent activation of GAF1 target genes, we examined the expression levels of GAF1 target genes in Col-0, *ga1-3*, and the *ga1-3 hac1* double mutant. Notably, the expression levels of GAF1 target genes were increased in *ga1-3* compared to Col-0. In contrast, DELLA-dependent activation of GAF1 target gene expression in the *ga1-3* disappeared in the *ga1-3 hac1* double mutant (Fig. 2n). Overall, these results support that HAC1 is involved in DELLA-dependent activation of GAF1 target genes.

### Recruitment of HAC1 to GAF1 target promoters in a DELLA-dependent manner

**Figure 3:**
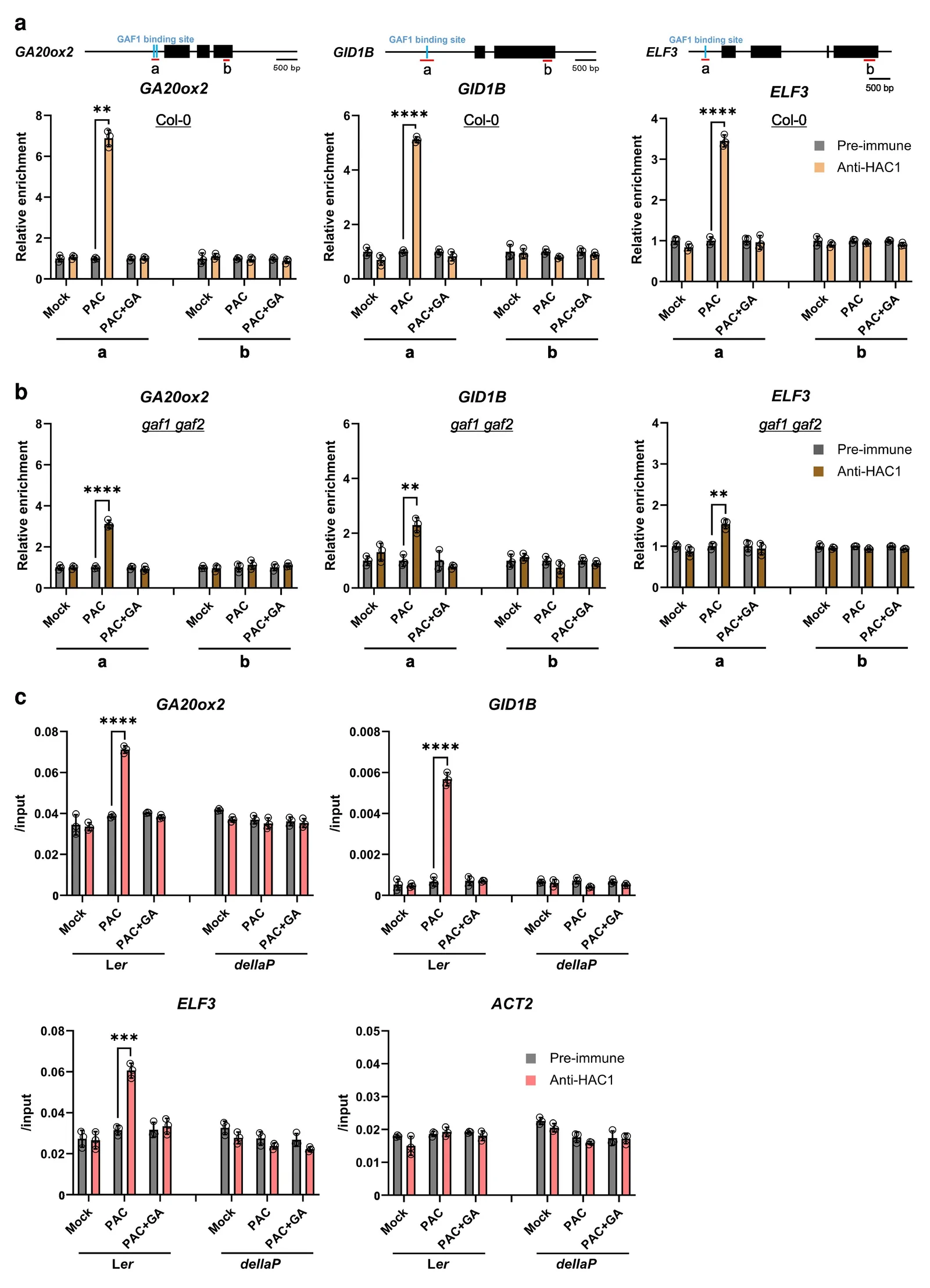
PAC-induced HAC1 recruitment to GAF1-target promoters is dependent on DELLA. a–c) ChIP-qPCR analysis of HAC1 levels on the promoter and coding regions of *GA20ox2*, *GID1B*, and *ELF3* in Col-0 (a), *gaf1 gaf2* double mutant (b), or promoter region of *GA20ox2*, *GID1B*, and *ELF3* in L*er* and *dellaP* (c). Schematic diagrams of *GA20ox2*, *GID1B*, *ELF3*, *UBQ11*, and PCR amplicons are indicated as red horizontal lines. 7-d-old Col-0 (a), *gaf1 gaf2* double mutant (b), or L*er* and *dellaP* (c) seedlings were treated with or without 1 μM PAC or 10 μM GA_3_ for 7 d. ChIP assays were performed with pre-immune or anti-HAC1 antibodies. The co-precipitated level of each DNA fragment was quantified using real-time PCR and normalized to the input DNA. Error bars indicate SD of 3 technical replicates (*n* = 3). Asterisks indicate significant difference compared with the pre-immune control, based on Student's *t* test (**P < 0.01, ***P < 0.001, ****P < 0.0001).

Since DELLA interacts with both HAC1 and GAF1, we tested whether HAC1 forms a complex with GAF1 in a DELLA-dependent manner using a yeast three-hybrid (Y3H) assay. The results showed that HAC1 did not directly interact with GAF1 but was implied to form a complex with GAF1 in a GAI-dependent manner in yeast (Figure S8). Next, we performed the chromatin immunoprecipitation (ChIP) analysis using an anti-HAC1 antibody to examine whether HAC1 is recruited to GAF1 target genes in a DELLA-dependent manner in vivo. Consequently, HAC1 was recruited to the GAF1 target promoters under GA-deficient conditions induced by PAC treatment (Fig. 3a). Furthermore, to test whether HAC1 recruitment is dependent on GAF1, we examined HAC1 recruitment in the *gaf1 gaf2* double mutant. HAC1 recruitment was not completely abolished in the *gaf1 gaf2* double mutant, but it was attenuated compared to Col-0 (Fig. 3b). Next, to examine whether the recruitment of HAC1 to GAF1 target promoters is DELLA-dependent, we performed a ChIP assay for HAC1 using the *della pentuple* mutant (*dellaP*) in the L*er* background. In L*er*, PAC treatment promoted the recruitment of HAC1 to GAF1 target promoters. Nevertheless, this PAC-induced recruitment of HAC1 was completely abolished in the *dellaP* (Fig. 3c).

**Figure 4:**
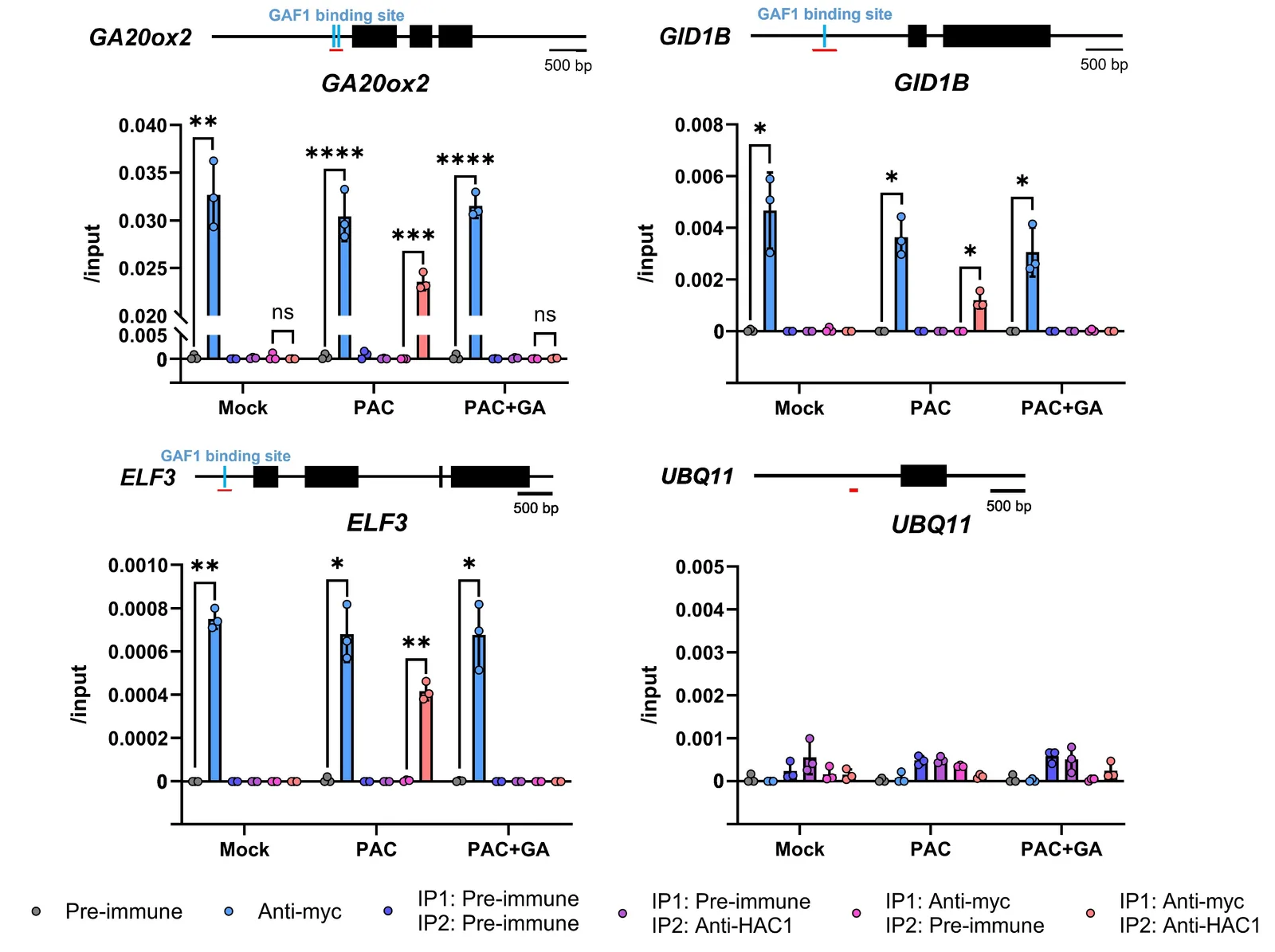
Co-localization of GAF1 and HAC1 under GA-deficient conditions. Re-ChIP-qPCR analysis of GAF1 and HAC1 levels at the promoter regions of *GA20ox2*, *GID1B*, *ELF3*, and *UBQ11* in *35S:4×myc-GAF1* transgenic plants. *UBQ11* was used as a negative control. Schematic diagrams of *GA20ox2*, *GID1B*, *ELF3*, *UBQ11*, and PCR amplicons are indicated as red horizontal lines. 7-d-old *35S:4×myc-GAF1* transgenic seedlings were treated with or without 1 μM PAC or 10 μM GA_3_ for 7 d. First ChIP (IP1) was performed with pre-immune or anti-myc antibodies. Second ChIP (IP2) was performed with pre-immune or anti-HAC1 antibodies. The co-precipitated level of each DNA fragment was quantified using real-time PCR and normalized to the input DNA. Error bars indicate the SD of 3 technical replicates (*n* = 3). Asterisks indicate significant differences by Student's *t-test* (ns; not significant, *P < 0.05, **P < 0.01, ***P < 0.001, ****P < 0.0001).

Furthermore, we conducted a re-ChIP assay to investigate whether GAF1 and HAC1 form a complex via DELLA in planta. Briefly, *35S:4×myc-GAF1* transgenic plants were treated with PAC and/or GA, and the first ChIP (IP1) was conducted using an anti-myc antibody, followed by a second ChIP (IP2) using an anti-HAC1 antibody. The results indicated that GAF1 constitutively binds to target promoters, whereas HAC1 recruitment to the GAF1 target promoters occurs only under GA-deficient conditions (Fig. 4). These results strongly support that HAC1 is recruited to GAF1 target promoters in a DELLA-dependent manner.

### HAC1 is involved in the transcriptional activity of DELLA via histone H3 acetylation

**Figure 5:**
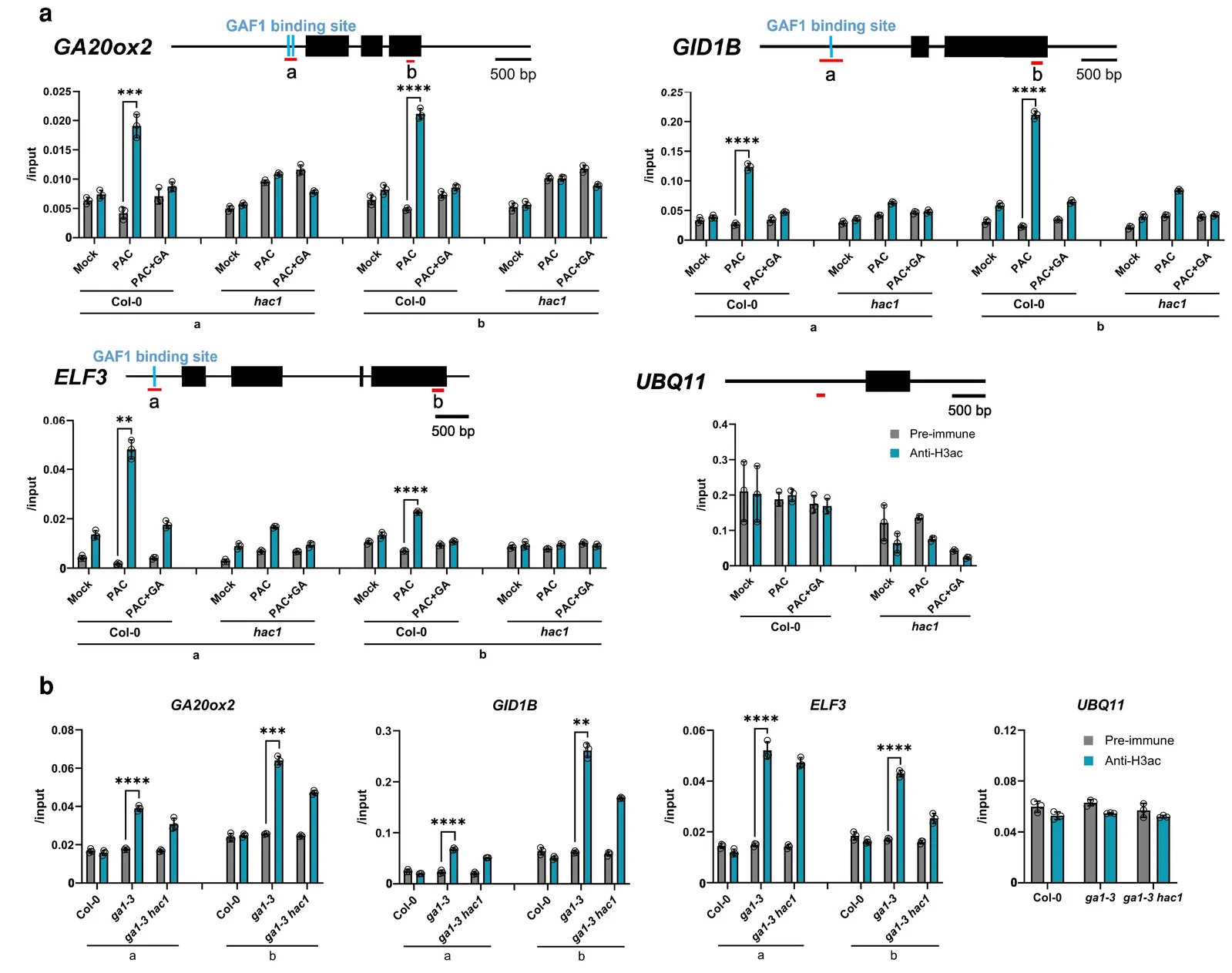
Enhancement of histone H3 acetylation at GAF1-target genes under GA-deficient conditions but attenuated by loss of HAC1. a, b) ChIP-qPCR analysis of histone H3ac levels on the promoter and coding regions of *GA20ox2*, *GID1B*, *ELF3*, and *UBQ11* in Col-0 and *hac1* mutant (a) and Col-0, *ga1-3*, and *ga1-3 hac1* double mutant (b). *UBQ11* was used as a negative control. Schematic diagrams of *GA20ox2*, *GID1B*, *ELF3*, *UBQ11*, and PCR amplicons are indicated as red horizontal lines. 7-d-old Col-0 and *hac1* seedlings were treated with or without 1 μM PAC or 10 μM GA_3_ for 7 d (a), or Col-0, *ga1-3*, and *ga1-3 hac1* double mutants were grown on half-strength MS agar medium for 14 d (b). ChIP assays were performed with pre-immune or anti-H3ac antibodies. The co-precipitated level of each DNA fragment was quantified using real-time PCR and normalized to the input DNA. Error bars indicate the SD of 3 technical replicates (*n* = 3). Asterisks indicate significant difference compared with the pre-immune control, based on Student's *t*-test (**P < 0.01, ***P < 0.001, ****P < 0.0001).

The GAF1–DELLA–HAC1 complex may acetylate histone H3 and promote the expression of GAF1 target genes. Therefore, we investigated the contribution of HAC1 to the transcriptional activation of target genes by the GAF1–DELLA complex. ChIP analysis using an anti-acetyl-histone H3 antibody (anti-H3ac) revealed increased acetylation levels of histone H3 in both the promoter and coding regions of GAF1-DELLA target genes in PAC-treated Col-0 plants. However, GA treatment attenuated PAC-induced increases the acetylation levels of histone H3 (Fig. 5a). In contrast, PAC-dependent increase in the histone H3 acetylation levels of GAF1 target genes was attenuated in the *hac1* mutant (Fig. 5a). Moreover, histone H3 acetylation levels showed a significant increase in the *ga1-3* but attenuated in the *ga1-3 hac1* double mutant (Fig. 5b). Overall, these results indicate that DELLA promotes the transcription of target genes by facilitating the acetylation of histone H3 in chromatin via HAC1.

To investigate whether the transcriptional activity of DELLA requires the HAC1 protein, we compared the transcriptional activities of DELLA in mesophyll protoplasts from Col-0 and *the hac1* mutant. Resultingly, GAI exhibited strong transcriptional activity in the mesophyll of Col-0, whereas this effect was weakened in mesophyll protoplasts of the *hac1* mutant, despite the BD-GAI protein accumulating to higher levels (Figure S9). Collectively, these results support the hypothesis that HAC1 is involved in the transcriptional activity of DELLA.

Furthermore, we examined *HAC1* expression levels in the presence or absence of GA and found that *HAC1* expression was downregulated following GA treatment, but upregulated following PAC treatment (Figure S10a). Importantly, the *HAC1* promoter was repressed by the GAF1–TPR4 complex and was activated by the GAF1–GAI complex, respectively (Figure S10b), which indicated that the GAF1 complex regulated *HAC1*.

### GAF1–DELLA complex directly activates the promoter of novel GAF1 target gene *ERF2*

**Figure 6:**
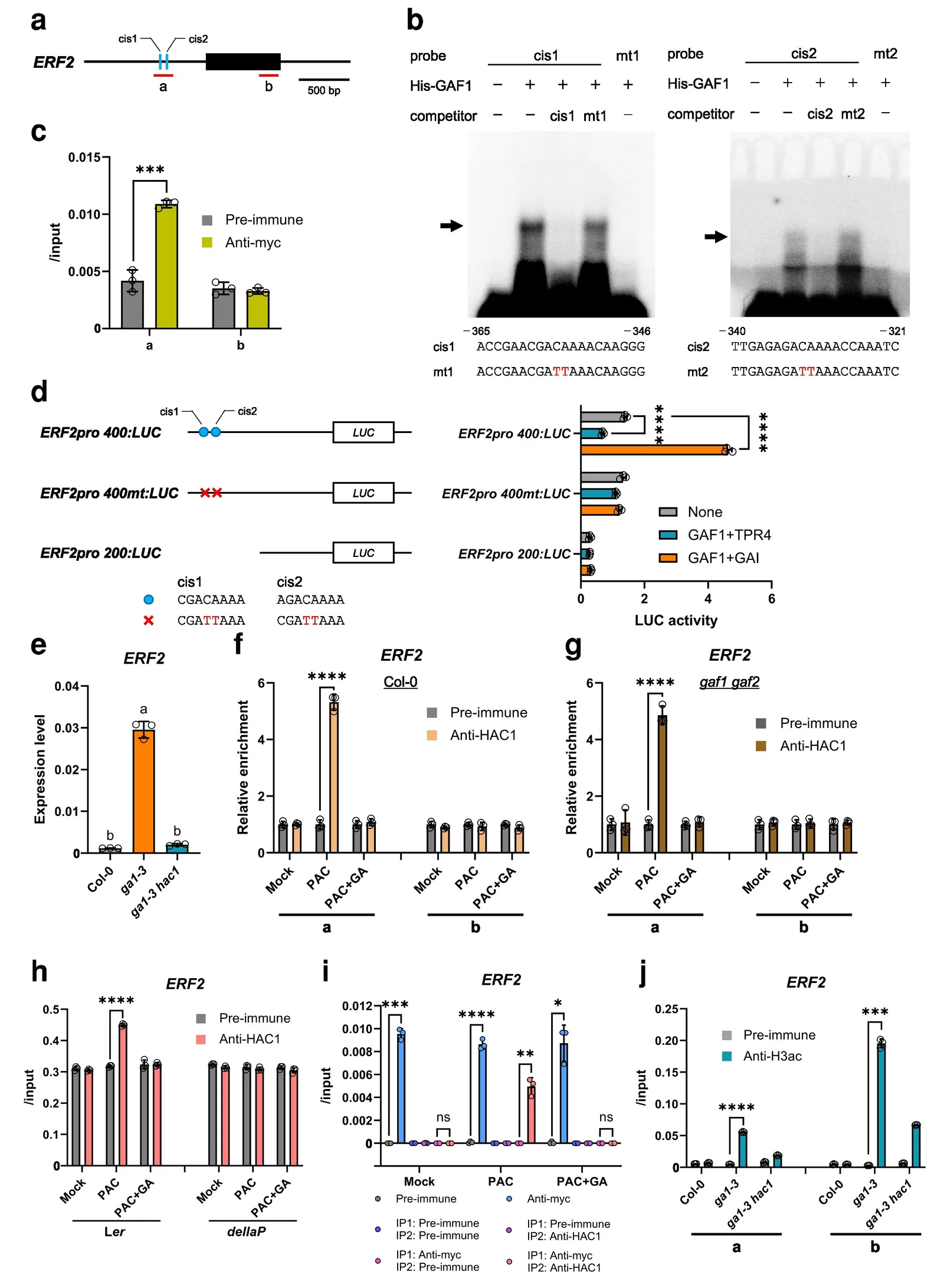
*ERF2* expression is regulated by the GAF1 complex and is subject to histone H3 acetylation mediated by HAC1. a) Schematic diagrams of *ERF2* and PCR amplicons, indicated by red horizontal lines, used for ChIP-qPCR. The black box indicates the exon region. b) Electrophoretic mobility shift assay (EMSA) of recombinant 6×His-GAF1 with a DNA probe containing cis1 (−365 to −346, lanes 1–4) or mtcis1 (lane 5) (left) and cis2 (−340 to −321, lanes 1-4) or mtcis2 (lane 5) (right) in the *ERF2* promoter region. Red letters indicate mutated bases. The competitors were applied using a 500-fold concentration of unlabeled wild-type and mutated probes. c) ChIP-qPCR analysis of 4×myc-GAF1 binding to the ID1-cis motifs in *ERF2* promoter. *35S:4×myc-GAF1* transgenic plant was grown on half-strength MS agar medium for 14 d. ChIP assays were performed with pre-immune or anti-myc antibodies. The co-precipitated level of each DNA fragment was quantified using real-time PCR and normalized to the input DNA. Error bars indicate SD of 3 technical replicates (*n* = 3). d) Regulation of the wild-type and mutated *ERF2* promoter by the GAF1 complex in mesophyll protoplasts. Each effector was introduced into protoplasts derived from Col-0 mesophyll cells with the reporter, and LUC/rLUC activity was measured after 20 h of culture. Error bars indicate SD of 3 biological replicates (*n* = 3). e) Expression level of *ERF2* in 14-d-old Col-0, *ga1-3*, and *ga1-3 hac1* seedlings. The expression levels of the target genes were normalized to that of *UBQ11*. Error bars indicate the SD of 3 biological replicates (*n* = 3). Different letters above the bars indicate significant differences (one-way ANOVA: Tukey–Kramer's multiple comparisons test, *P* < 0.05). f–h) ChIP-qPCR analysis of HAC1 levels on the promoter and coding regions of *ERF2* in Col-0 (f), *gaf1 gaf2* double mutant (g), or promoter region of *ERF2* in L*er* and *dellaP* (h). Schematic diagrams of *ERF2* and PCR amplicons are indicated as red horizontal lines. 7-d-old Col-0 (f), *gaf1 gaf2* double mutant (g), or L*er* and *dellaP* (h) seedlings were treated with or without 1 μM PAC or 10 μM GA_3_ for 7 d. ChIP assays were performed with pre-immune or anti-HAC1 antibodies. The co-precipitated level of each DNA fragment was quantified using real-time PCR and normalized to the input DNA. Error bars indicate the SD of 3 technical replicates (*n* = 3). i) Re-ChIP-qPCR analysis of GAF1 and HAC1 levels on the promoter region of *ERF2* in *35S:4×myc-GAF1* transgenic plants. 7-d-old *35S:4×myc-GAF1* transgenic seedlings were treated with or without 1 μM PAC or 10 μM GA_3_ for 7 d. First ChIP (IP1) was performed with pre-immune or anti-myc antibodies. Second ChIP (IP2) was performed with pre-immune or anti-HAC1 antibodies. The co-precipitated level of each DNA fragment was quantified using real-time PCR and normalized to the input DNA. Error bars indicate SD of 3 technical replicates (*n* = 3). j) ChIP-qPCR analysis of histone H3ac levels on the promoter and coding regions of *ERF2* in Col-0, *ga1-3*, and *ga1-3 hac1* double mutant. Plants were grown on half-strength MS agar medium for 14 d. ChIP assays were performed with pre-immune or anti-H3ac antibodies. The co-precipitated level of each DNA fragment was quantified using real-time PCR and normalized to the input DNA. Error bars indicate SD of 3 technical replicates (*n* = 3). Asterisks indicate significant differences based on Student's *t*-test (ns; not significant, *P < 0.05, **P < 0.01, ***P < 0.001, ****P < 0.0001).

In our previous study, the overexpression of GAF1ΔPAM, a constitutive repressor lacking the PAM domain required for interaction with DELLA, partially restored the dwarf phenotype of *ga1-3* (Fukazawa et al. 2014). This suggests that GAF1 target genes may include growth repressor genes. Previously, we identified GAF1 target genes, including *GA20ox2*, *GID1B*, and *ELF3* (Fukazawa et al. 2014, 2017, 2021); however, these genes could not sufficiently explain GAF1-DELLA-dependent growth inhibition. *ETHYLENE RESPONSE FACTOR2* (*ERF2*) is a candidate target gene of GAF1 because *ERF2* expression is downregulated by induced GAF1 overexpression (Fukazawa et al. 2021). Additionally, *ERF2* is induced by ethylene and JA (Brown et al. 2003; McGrath et al. 2005). Our recent findings indicate that JA inhibits plant growth via the reduction of endogenous GA and the accumulation of DELLA (Fukazawa et al. 2023). Therefore, *ERF2* is a growth repressor candidate that is regulated by GAF1–DELLA complex. To investigate whether the GAF1 complex regulates *ERF2* expression, we searched for a GAF1 binding sequence (TTTGTCGTATT or TTTTGTCG) (Kozaki et al. 2004; Fukazawa et al. 2014) in the *ERF2* promoter. Two GAF1 binding sequences were identified from the −359 to −352 bp region (cis1; +1 indicates start codon) and from the −335 to −328 bp region (cis2) on the *ERF2* promoter (Fig. 6a). Additionally, electrophoretic mobility shift assay (EMSA) demonstrated that the GAF1 protein bound to both the cis1 and cis2 regions of the *ERF2* promoter (Fig. 6b). To confirm the binding of GAF1 to the *ERF2* promoter in vivo, we performed a ChIP assay using a transgenic plant expressing myc-tagged GAF1. The ChIP assay showed that GAF1 bound to the cis1 and cis2 regions of the *ERF2* promoter in vivo (Fig. 6c). Furthermore, to investigate whether the GAF1 complex regulates the *ERF2* promoter, we performed a transient analysis using Col-0 mesophyll protoplasts. Notably, the *ERF2* promoter was repressed by the GAF1–TPR4 complex but activated by the GAF1–GAI complex. In contrast, mutated and deleted *ERF2* promoters (lacking GAF1 binding sites) were not regulated by the GAF1 complex (Fig. 6d). Overall, we identified *ERF2* as a novel GAF1 target gene.

### *ERF2* expression is regulated by DELLA–HAC1 complex through the histone H3 acetylation

To investigate whether HAC1 is involved in regulating *ERF2* expression, we compared *ERF2* expression levels in Col-0, *ga1-3*, and the *ga1-3 hac1* double mutant. *ERF2* expression level increased in *ga1-3* compared with that in Col-0. In contrast, *ERF2* expression level did not display any increase in the *ga1-3 hac1* double mutant (Fig. 6e). Moreover, we examined whether HAC1 is recruited to the GAF1 binding sites of the *ERF2* promoter. Notably, HAC1 is recruited to the *ERF2* promoter region under GA-deficient conditions induced by PAC treatment (Fig. 6f). We investigated whether HAC1 recruitment under GA-deficient conditions depends on GAF1 by using the *gaf1 gaf2* double mutant and found that HAC1 recruitment was slightly attenuated compared to Col-0 (Fig. 6g). To investigate whether HAC1 recruitment to the *ERF2* promoter is DELLA-dependent, we conducted ChIP analysis using an anti-HAC1 antibody. In the *dellaP*, HAC1 was not recruited under GA-deficient conditions (Fig. 6h). Re-ChIP analysis was conducted to examine GAF1 binding and HAC1 recruitment to the *ERF2* promoter. The results demonstrated that GAF1 binds constitutively to the *ERF2* promoter, whereas HAC1 is recruited only under GA-deficient conditions (Fig. 6i). These results indicate that HAC1 is recruited in a DELLA-dependent manner to the GAF1-bound regions on the *ERF2* promoter. Additionally, we examined the histone H3 acetylation levels in the *ERF2* promoter and coding region and found that histone H3 acetylation levels exhibited a significant increase in the *ga1-3* but were attenuated in the *ga1-3 hac1* double mutant (Fig. 6j). Collectively, these results indicate that the GAF1–DELLA complex regulates *ERF2* transcription via HAC1-mediated histone H3 acetylation.

### *ERF2* acts as a growth repressor downstream of GAI

**Figure 7:**
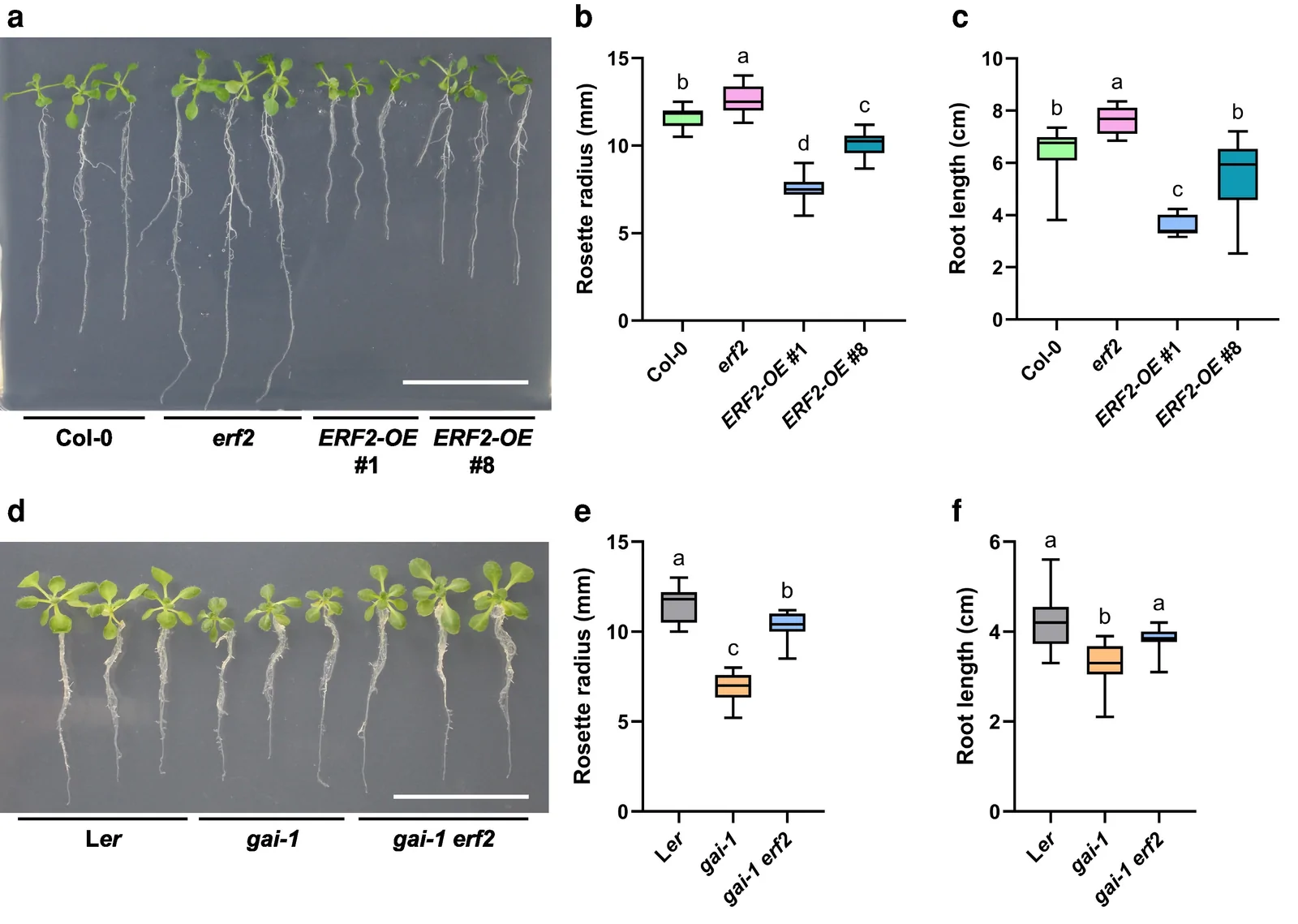
ERF2 acts as a growth repressor downstream of GAI. a) Phenotypes of Col-0, *erf2* mutant, *ERF2-OE* #1, and #8 seedlings grown on half-strength MS agar medium for 14 d. Scale bar = 3 cm. b, c) Rosette radius (b) and root length (c) of Col-0, *erf2* mutant, *ERF2-OE* #1, and #8 seedlings grown on half-strength MS agar medium for 14 d; *n* = 12 biologically independent samples. d) Phenotypes of L*er*, *gai-1*, and *gai-1 erf2* seedlings grown on half-strength MS agar medium for 14 d. Scale bar = 3 cm. e, f) Rosette radius (e) and root length (f) of L*er*, *gai-1*, and *gai-1 erf2* seedlings grown on half-strength MS agar medium for 14 d; *n* ≥ 11 biologically independent samples. In all boxplots shown in b, c, and e, f), center lines and box edges are medians and the IQR, respectively. Whiskers extend to the lowest and highest data points within 1.5 times the IQR below and above the lower and upper quartiles, respectively. Different letters above the bars indicate statistically significant differences (one-way ANOVA: Tukey–Kramer's multiple comparisons test, *P* < 0.05).

To examine the role of *ERF2* in plant growth, we generated an *erf2* mutant using the CRISPR-Cas9 system and *ERF2*-overexpressing lines (*ERF2-OE*) (Figure S11). The *erf2* mutant exhibited an expanded rosette radius and an elongated root length. In contrast, rosette radius and root length were smaller in the *ERF2-OE* than in Col-0 (Fig. 7a–c). Overall, these results indicate that *ERF2* acts as a growth repressor gene. Considering that *ERF2* is a DELLA-regulated growth repressor gene, the growth levels of the *erf2* mutant and *ERF2-OE* were examined under conditions of DELLA accumulation. PAC-induced growth inhibition of the rosette radius was attenuated in the *erf2* mutant, whereas this growth inhibition was enhanced in the *ERF2-OE* compared with that in Col-0 (Figure S12).

Furthermore, we investigated whether *ERF2* affects germination and flowering. All plants exhibited similar germination rates in the Col-0, *erf2*, and *ERF2-OE* lines (Figure S13a, b). Col-0 and *erf2* mutant flowered at the same time, but the *ERF2-OE* exhibited a late-flowering phenotype (Figure S13c–f).

Finally, to investigate the genetic relationship between DELLA and ERF2, we crossed the *gai-1* mutant with the *erf2* mutant and analyzed the resulting phenotypes. The *gai-1* mutant exhibits a dwarf phenotype, whereas the *gai-1 erf2* double mutant displayed almost complete restoration of rosette and root growth (Fig. 7d–f). The *gai-1 erf2* double mutant is in a heterogeneous background. We confirmed that restoration of the *gai-1* phenotype depends on the presence of the *erf2* mutation (Figure S14). These results indicate that ERF2 functions downstream of GAI as a growth repressor.

## Discussion

**Figure 8:**
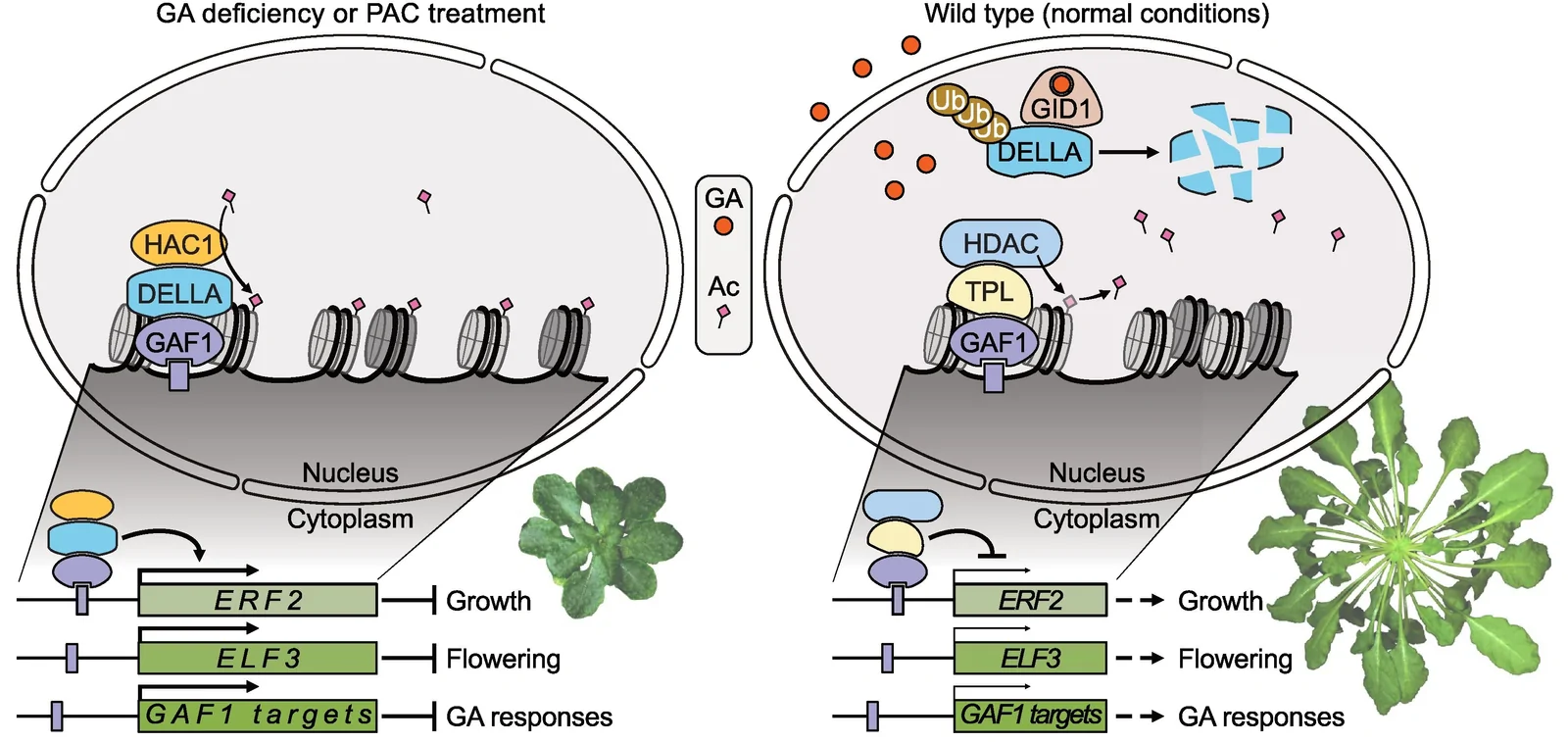
Working model of HAC1-mediated transcriptional activation by DELLA. In the absence of gibberellin (GA), DELLA accumulates and recruits HAC1 to acetylate histone H3 on GAF1 target chromatin, thereby facilitating transcription. In contrast, when DELLA is degraded in the presence of GA, GAF1 interacts with TPL to repress target gene transcription. *ERF2* was identified as a novel target gene of GAF1. Upon accumulation of DELLA, the GAF1–DELLA–HAC1 complex promotes *ERF2* expression, which inhibits plant growth. Regular arrows and blunt-ended arrows indicate positive and negative regulation, respectively. Ac, acetyl group; Ub, ubiquitin.

Our study revealed that DELLA-dependent transcriptional activation of the GAF1 target gene requires HAC1-mediated histone H3 acetylation (Fig. 8). DELLA and TPL/TPR act as transcriptional co-activators and co-repressors with GAF1, respectively (Fukazawa et al. 2014). The transcriptional activity of the GAF1 complex depends on GAF1 interaction factors such as DELLA and TPL/TPR. Considering that DELLA proteins are degraded by GA, GA controls the transcriptional activity of the GAF1 complex (Fukazawa et al. 2014). Additionally, TPL/TPR represses transcription by recruiting HDAC (Krogan et al. 2012; Yusuf et al. 2025). In this study, we showed that HAC1, a HAT, interacts with DELLA and that it is required for transcriptional activation by DELLA. Moreover, DELLA and TPL/TPR may recruit HAC1 and HDAC to the promoters of target genes, and acetylate and deacetylate histone H3, respectively (Fig. 8). Although our study focused on transcriptional activation by DELLA, DELLA may repress some genes (Park et al. 2017; Huang et al. 2023, 2024). For example, RGA represses the expression of the flowering time regulator SQUANOSA PROMOTER BINDING PROTEIN-LIKE15 (SPL15) target genes by inhibiting the interaction between SPL15 and MED18 (Hyun et al. 2016). Additionally, SLR1, a rice DELLA protein, interacts with POLYCOMB-REPRESSIVE COMPLEX2 (PRC2) and HISTONE DEACETYLASE702 (HDA702) and represses the expression of target genes through the repressive mark trimethylation of lysine 27 of histone H3 (H3K27me3) and histone deacetylation (Li et al. 2023). Although DELLA proteins activate or repress the expression of target genes, the mechanisms underlying both functions remain unknown.

Herein, we performed a comprehensive screening of DELLA-interacting proteins using the AirID system and identified biotinylated proteins as candidates for DELLA-interacting proteins (Table S1). Importantly, this approach enables screening under in vivo-like conditions, thereby reflecting the intracellular localization of DELLA and its interacting factors. Although the study by Kim et al. (2023) utilized TurboID, it demonstrated that the BRASSINOSTEROID-INSENSITIVE2 (BIN2) kinase-TurboID can effectively and specifically capture phosphorylation substrates (Kim et al. 2023), thereby highlighting the utility of proximity biotin labeling for in vivo. Considering many proteins form complexes with other proteins in vivo, analyzing the interacting proteins is useful for understanding their functions. Overall, the screening of DELLA-interacting proteins under in vivo conditions, as in the present study, provides key insights into the function of DELLA proteins.

MED15 has been identified as a factor involved in DELLA-mediated transcriptional activation (Hernández-García et al. 2024). Collectively, these findings suggest that HAC1 promotes histone H3 acetylation on the target chromatin, which relaxes the chromatin structure and facilitates the recruitment of the MED complex and RNA polymerase II through MED15, thereby enhancing the transcription of target genes. However, both HAC1 and MED15 possess a KIX domain (Thakur et al. 2013) and interact with the N-terminal region of DELLA (Fig. 1e) (Hernández-García et al. 2024). However, Ohama et al. (2025) demonstrated that MED15ΔKIX, which lacks the KIX domain, can interact with RGA. Furthermore, we demonstrated that HAC1ΔKIX and nHAC1ΔKIX interacts with DELLA (Figures S4e, S4f, S5b). However, it remains unclear whether HAC1 and MED15 compete with DELLA, or whether DELLA possesses a mechanism that selectively utilizes interaction regions of HAC1 and MED15 other than their KIX domain.

HAC1 was recruited to GAF1 target promoters under GA-deficient conditions in Col-0 (Figs. 3a, 6f), but it was not completely abolished in the *gaf1 gaf2* double mutant (Figs. 3b, 6g). These results suggest the existence of factors that are functionally redundant with GAF1. The *Arabidopsis* genome encodes 16 IDD TFs (Colasanti et al. 2006), and AtIDD3, 4, 5, 9, and 10 have been reported to interact with AtDELLAs and promote the transcription of target genes (Yoshida et al. 2014). Furthermore, IDD3 bind to a core sequence (AGACAA) similar to the GAF1-binding motif (Yoshida et al. 2014), suggesting that HAC1 might be recruited to target genes via DELLA and IDD TFs other than GAF1 and GAF2.

HAC1 is recruited to the GAF1 target promoters, but not to coding regions (Figs. 3 and 6f, 6g). However, acetylation of histone H3 occurs in both promoter and coding regions (Figs. 5 and 6j). In a previous study, HAC1 was recruited to the *PATHOGENESIS-RELATED 1* (*PR1*) and *PR2* promoter regions in response to the synthetic SA analog INA and pathogen infection, but not to their coding regions. However, histone H3 acetylation extended into the coding regions (Jin et al. 2018). Furthermore, CONSTANS (CO), who play a key role in the photoperiod-dependent flowering pathway, recruited HAC1 to the promoter regions of 2 key floral pathway integrators, *FLOWERING LOCUS T* (*FT*) and *SUPPRESSOR OF OVEREXPRESSION OF CONSTANS 1* (*SOC1*), whereas histone H3 acetylation extended into the coding region of *SOC1* (Liang et al. 2025). Our findings are consistent with those of previous studies; however, the mechanism of HAC1 recruitment to the promoter region, which acetylates histone H3 in the coding region, remains unclear. One plausible explanation is that histone acetylation spreads through bromodomain-containing proteins. A subset of HATs contains bromodomains, which are protein modules that recognize acetylated lysine residues on histone tails (Filippakopoulos and Knapp 2014). This structural feature enables these HATs to function not only as “writers” of histone acetylation but also as “readers” of pre-existing acetylation marks. Through this dual functionality, bromodomain-containing HATs can be recruited to chromatin regions that are already acetylated and further propagate histone acetylation in a self-reinforcing manner. In *Arabidopsis*, GCN5/HAG1, belonging to the GNAT family and HAF1 and HAF2, belonging to the TAFII250 family, are HATs containing a bromodomain (Rao et al. 2014). GCN5/HAG1 was also detected in the AirID-based screening we conducted, thereby suggesting that it functions in the vicinity of GAI and RGA (Table S1). We propose that HAC1 is recruited to GAF1 target promoters in a DELLA-dependent manner and leads to the establishment of local histone acetylation. This initial acetylation may subsequently be recognized by bromodomain-containing HATs, such as GCN5/HAG1-associated complexes, which can bind acetylated histones and further promote histone acetylation. This mechanism could facilitate the spreading of histone acetylation into coding regions and ultimately establish broad acetylation associated with active transcription.

Compared with PAC-treated Col-0 and *ga1-3*, the reduction in expression of GAF1-target genes and in histone H3 acetylation levels was particularly pronounced in PAC-treated *hac1* mutant and the *ga1-3 hac1* double mutant (Figs. 2d, 2n, 5 and 6e, 6j), supporting the conclusion that HAC1 plays a major role in GAF1–DELLA complex-mediated transcriptional activation. However, the DELLA-dependent activation of GAF1 target genes in PAC-treated Col-0 and *ga1-3* was not fully eliminated in the PAC-treated *hac1* mutant and the *ga1-3 hac1* double mutant (Figs. 2d, 2n, 5 and 6e, 6j), which may be attributed to functionally redundant factors. *Arabidopsis* has 5 members of the HAC family: HAC1, HAC2, HAC4, HAC5, and HAC12. Among them, HAC2 lacks HAT activity (Bordoli et al. 2001), whereas the other 4 members exhibit similar expression patterns in flowers, shoot apical meristems, leaves, and roots (Han et al. 2007; Li et al. 2014). Moreover, the KIX domain, which interacts with DELLA, is conserved among the 4 HACs (Thakur et al. 2013; Li et al. 2014). Analysis of multiple mutants with various combinations of the 4 HACs showed that the *hac1 hac5* double mutant exhibited a more severe growth defect than the *hac1* single mutant, thereby suggesting that HAC5 was functionally most redundant with HAC1 (Li et al. 2014). HAC12 was also detected in the AirID-GAI and -RGA screens we performed (Table S1), suggesting that HAC5 and HAC12 may function redundantly with HAC1.

Notably, the late-flowering phenotype of the *ga1-3* was partially restored in the *ga1-3 hac1* double mutant (Fig. 2j–m). Additionally, the expression level of *ELF3*, a GAF1-DELLA-regulated flowering repressor gene (Fukazawa et al. 2021), was upregulated in the *ga1-3,* but this increase was attenuated in the *ga1-3 hac1* double mutant (Fig. 2n). Furthermore, the low germination rate and growth inhibition phenotypes of the *ga1-3* were also partially restored in the *ga1-3 hac1* double mutant (Fig. 2e–i). Moreover, GAF1*Δ*PAM overexpression partially restored the low germination, dwarf phenotype, and late-flowering in the *ga1-3* (Fukazawa et al. 2014). Overall, these results indicate the existence of germination- and growth-repressor genes that are activated by the GAF1–DELLA complex. Specifically, we identified *ERF2* as a growth repressor that is regulated by the GAF1–DELLA complex via HAC1-mediated histone H3 acetylation (Fig. 6). However, since ERF2 is not involved in germination (Figure S13a, b), future studies should focus on identifying germination-repressor genes regulated by the GAF1–DELLA–HAC1 complex.

Importantly, plant growth and defense activities involve trade-offs, with plants inhibiting growth to activate defense responses under stress conditions. DELLA protein accumulation is promoted by various stress–responsive plant hormones such as JA (Yang et al. 2012; Fukazawa et al. 2023), SA (Yu et al. 2022), and abscisic acid (ABA) (Achard et al. 2006). Under stress conditions, such as herbivore attack, pathogen infection, drought, or salt stress, these plant hormones are synthesized and lead to the accumulation of DELLA proteins. Therefore, the GAF1–DELLA complex is considered to be formed and subsequently promotes the expression of *ERF2* and other growth-repressor genes through HAC1-mediated histone H3 acetylation, thereby inhibiting plant growth. A comprehensive understanding of DELLA-mediated transcriptional regulation and the downstream genes may provide insight for controlling plant growth artificially and for breeding crops that do not exhibit growth inhibition under stress conditions.

## Methods

### Plant materials and growing conditions

*A. thaliana* [Columbia (Col-0) ecotype] plants were grown on half-strength Murashige and Skoog (MS) medium [1.0% sucrose, 0.05% 2-(*N*-morpholino) Ethanesulfonic Acid (MES) (pH 5.8), 9 mM Thiamine Hydrochloride, 40 mM Nicotinic Acid, 2.4 mM Pyridoxine Hydrochloride, 0.8% Agar] in a growth chamber at 22 °C under a 16 h light/8 h dark cycle. The *hac1* (SALK_082118) mutant was obtained from the Arabidopsis Biological Resources Center (ABRC) at Ohio State University. The *gaf1 gaf2* double mutant and *35S:4×myc-GAF1* transgenic plant were described previously (Fukazawa et al. 2014). The *hac1* and *erf2* mutants are in the Col-0 background, while *dellaP* and *gai-1* are in the Landsberg *erecta* (L*er*) ecotype background. The GA-deficient mutant *ga1-3* was originally isolated in the L*er* but has since been introgressed into a Col-0 background by 6 rounds of backcrossing (Tyler et al. 2004). The *ga1-3 hac1* double mutant was generated by crossing the *ga1-3* and *hac1* mutants. The *gai-1 hac1* and *gai-1 erf2* double mutants were generated by crossing the *gai-1* and *hac1* or *erf2* mutants. To generate the *erf2* mutant, *ERF2* guide RNA1 (gRNA1; +334 to +356 bp) and gRNA2 (+49 to +71 bp) were cloned into the *Aar*I site of the pKAMA-ITACHI vector (Tsutsui and Higashiyama 2017). To generate transgenic plants overexpressing *ERF2*, *ERF2* was cloned into the *Not*I-*Xho*I sites of the binary pBIJ4 vector containing a CaMV 35S promoter with a viral translation enhancer, Ω sequence, as described previously (Fukazawa et al. 2010). Additionally, *Agrobacterium tumefaciens*-mediated *Arabidopsis* transformation was performed using the floral dip method (Clough and Bent 1998). The primers used for cloning and genotyping are listed in Table S2.

### Cloning genes

Template cDNAs or genomic DNAs were prepared from wild-type (Col-0) leaves for gene cloning. Target sequences were amplified using a Mastercycler Nexus GSX1 (Eppendorf, Hamburg, Germany) and cloned into a pJ4 vector (Fukazawa et al. 2010) or p-less vector (Fukazawa et al. 2017) using the Ligation high ver.2 (TOYOBO Co., Ltd. Osaka, Japan) system or seamless ligation cloning extract (SLiCE) system (Motohashi 2015). The primers used for cloning are listed in Table S2.

### Transient expression in *Arabidopsis* T87 cultured cells

*AGIA-AirID* was cloned into the *Sal*I-*Kpn*I sites, and cDNA of *GAI* or *RGA* was cloned into the *Kpn*I-*Sac*I sites of the pJ4 vector carrying the CaMV 35S promoter with the Ω sequence (Fukazawa et al. 2000) as an effector. The primers used for cloning are listed in Table S2. *A. thaliana* T87 cultured cells (rpc00008) were obtained from the RIKEN BioResource Center and derived from the Columbia ecotype. The T87 cells were grown in a Jouanneau and Péaud-Lenoël (JPL) medium supplemented with 1 µM 1-naphthaleneacetic acid (NAA), and the culture was maintained at 22 °C under continuous light with rotary shaking at 120 rpm. Protoplasts were prepared from T87 cultured *Arabidopsis* cells, transfected as previously described (Fukazawa et al. 2014), and cultured under continuous light for 20 h at 22 °C.

### Immunoblot analysis

To investigate protein levels in T87 cultured cells transiently expressing AGIA-AirID-GAI or AGIA-AirID-RGA, the harvested cells were suspended in 1× sodium dodecyl sulfate (SDS) sample buffer [50 mM Tris-HCl (pH 6.8), 2% SDS, 0.1% bromophenol blue, 10% glycerol and 0.1 mM β-mercaptoethanol], lysed using a Handy Sonic UR-20P (Tomy Seiko), and boiled for 10 min at 95 °C. Thereafter, the proteins were separated using sodium dodecyl sulfate-polyacrylamide gel electrophoresis (SDS-PAGE) (10% acrylamide gel) and immunoblotted with anti-AGIA HRP Rabbit mAb (Yano et al. 2016) at 1:10,000 dilution, anti-biotin HRP-linked antibody (Kido et al. 2020) at 1:5,000 dilution, anti-GFP antibody (MBL International, Woburn, MA, United States. 598/Lot#076) at 1:5,000 dilution, anti-HAC1 antibody (PhytoAB, San Jose, CA, United States. PHY7924A/Lot#20d58C6a) at 1:3,000 dilution, anti-FLAG antibody (BioLegend, San Diego, CA, United States. 637301/Lot#B185581) at 1:5,000 dilution, anti-HA-tag mAb-HRP-DirecT antibody (MBL International, M180-7/Lot#008) at 1:5,000 dilution, or anti-GAI antibody (generated in rabbit) at 1:1,000 dilution.

### Mass spectrometry analysis of biotinylated peptides

T87 cultured cells transiently expressing AGIA-AirID-GAI or AGIA-AirID-RGA were incubated in 0.5 mM biotin-containing protoplast medium for 20 h at 22 °C and subsequently harvested. T87 cells treated with PEG without any plasmid were used as negative controls. Thereafter, the harvested cells were suspended in guanidine-TCEP buffer [6 M guanidine-HCl, 100 mM HEPES-NaOH pH 7.5, 10 mM Tris(2-carboxyethyl) phosphine hydrochloride (TCEP), 40 mM 2-chloroacetamide] and lysed using a Handy Sonic UR-20P. Biotinylated peptides were isolated from the cell lysates using Tamavidin 2-REV and analyzed by LC-MS/MS, as previously described (Motani and Kosako 2020).

### Y2H assay

The *HAC1* full-length CDS, *HAC1^1–710aa^* (*nHAC1*) and *HAC1^711–1741aa^* (*cHAC1*) were cloned into the *Bam*HI-*Pst*I sites of pGBDU vector (Clontech). The pGAD:RGA, GAI, RGL1, RGL2 and RGL3 were constructed as previously described (Fukazawa et al. 2014). The primers used for cloning are listed in Table S2. *Saccharomyces cerevisiae* PJ69-4A was co-transformed with bait and prey plasmids. Positive transformants grown on SD/-Ura/-Leu medium plates were cultured in SD/-Ura/-Leu liquid medium, adjusted to an O.D._600_ value of 0.1, and 2 μL were spotted on SD/-Ura/-Leu and SD/-Ura/-Leu/-His medium plates containing various concentrations (0 to 200 mM) of 3-amino-1,2,4-triazole (3-AT).

### BiFC assay

The *nHAC1* and *cHAC1* were cloned into the *Not*I-*Bgl*Ⅱ sites of pJ4:cYFP (Fukazawa et al. 2014). pJ4:nYFP-GAI was used as previously described (Fukazawa et al. 2014). The primers used for cloning are listed in Table S2. Each plasmid was transiently expressed in Col-0 mesophyll protoplasts, as described below. Transfected Col-0 mesophyll protoplasts were cultured for 20 h, and YFP fluorescence was observed using an LSM 5 Pascal confocal microscope (Zeiss, Oberkochen, Germany).

### Cell-free protein synthesis and AlphaScreen

The *nHAC1* and *nHAC1ΔKIX* were cloned into the *Xho*I-*Kpn*I sites of the pEU:MCS-bls vector (Cell-Free Sciences, Ehime, Japan), and *cHAC1* was cloned into the *Xho*I-*Kpn*I sites of the pEU:bls-MCS vector (Cell-Free Sciences). The *AtDELLAs* were cloned into the pEU:GW-FLAG vector (Nemoto et al. 2018). The *DHFR* was cloned into the pEU:MCS-FLAG vector (Nozawa et al. 2023). The primers used for cloning are listed in Table S2. AlphaScreen was performed as previously described (Kido et al. 2020; Shibuya et al. 2024).

### Co-IP assay

The 7-d-old *RGApro:GFP-RGA* transgenic seedlings were transferred to half-strength MS medium with or without 1 μM PAC or 10 μM GA_3_ and grown for 7 d. Subsequently, 0.5 g of seedlings was frozen and ground to a fine powder in liquid nitrogen before homogenizing in protein extraction buffer [50 mM Tris-HCl pH 8.0, 150 mM NaCl, 1% Triton X-100, 5 mM EDTA, 1× protease inhibitor cocktail (Roche, Indianapolis, IN, United States)]. The extracts were centrifuged at 15,500 g at 4 °C for 10 min to remove cellular debris and incubated with an anti-GFP antibody (MBL International, Woburn, MA, United States, 598/Lot#076). The protein-antibody complexes were precipitated using FG Protein G beads (Tamagawa Seiki Co., Ltd. Nagano, Japan. Lot#345SNENCPG2) overnight at 4 °C and then washed 3 times with extraction buffer. The immunoprecipitated proteins were eluted and analyzed by immunoblot analysis using rabbit polyclonal anti-GFP antibody (MBL International, Woburn, MA, United States. 598/Lot#076) and rabbit polyclonal anti-HAC1 antibody (PhytoAB, San Jose, CA, United States. PHY7924A/Lot#20d58C6a) as described above. A rabbit's pre-immune serum as a negative control. Quantitative analysis of the relative signal intensity was performed using ImageJ.

*FLAG* or *HA* were cloned into the *Not*I site, and cDNA of *GAI* or *nHAC1*, *nHAC1ΔKIX*, and *cHAC1* were cloned into the *Not*I-*Bgl*Ⅱ sites of the pJ4 vector carrying the CaMV 35S promoter with the Ω sequence (Fukazawa et al. 2000). The primers used for cloning are listed in Table S2. FLAG-GAI and HA-nHAC1, HA-nHAC1*Δ*KIX, or HA-cHAC1 were transiently co-expressed in T87 cultured cells as described above, and the cells were harvested 24 h after transfection. Total protein complexes were extracted from the harvested cells using a protein extraction buffer, and the extracts were incubated with an anti-FLAG antibody (BioLegend, San Diego, CA, United States, 637301/Lot#B185581) overnight at 4 °C. The protein–antibody complexes were precipitated using FG Protein G beads (Tamagawa Seiki Co., Ltd. Lot#345SNENCPG2) overnight at 4 °C and subsequently washed 3 times with extraction buffer. The immunoprecipitated proteins were eluted and analyzed by immunoblot analysis using anti-FLAG antibody (BioLegend, San Diego, CA, United States, 637301/Lot#B185581) and anti-HA-tag mAb-HRP-DirecT antibody (MBL International, M180-7/Lot#008) as described above.

### Gene expression analysis

Total RNA was isolated from seedlings using a Total RNA Extraction Kit (Plant) (RBC Bioscience, New Taipei City, Taiwan, Cat#YRP100). cDNA was synthesized from 500 ng of total RNA using the ReverTra Ace^TM^ qPCR RT Master Mix with gDNA Remover (TOYOBO Co., Ltd. Cat#FSQ-301) according to the manufacturer's instructions. Quantitative real-time PCR was performed in triplicate on a CFX connect Real-Time PCR Detection System (Bio-Rad Laboratories, Hercules, CA, United States) using GeneAce SYBR qPCR Mixa No Rox (NIPPON GENE, Tokyo, Japan, Cat#319-07703). The expression levels of target genes were normalized to that of *UBQ11* expression. The primers used for gene expression analysis are listed in Table S2.

### ChIP-qPCR, Re-ChIP-qPCR

ChIP experiments were performed as previously described (Fukazawa et al. 2023) with some modifications. For ChIP analysis, 7-d-old Col-0, *gaf1 gaf2* double mutant, and *hac1* mutant seedlings were transferred to half-strength MS medium with or without 1 μM PAC or 10 μM GA_3_ and grown for 7 d. Alternatively, 14-d-old Col-0, *ga1-3*, and *ga1-3 hac1* double-mutant seedlings, or 14-d-old *35S:4×myc-GAF1* transgenic seedlings (Fukazawa et al. 2014) were used. Briefly, approximately 0.5 g of each seedling was cross-linked in 1% (v/v) formaldehyde under vacuum for 20 min and then incubated for 10 min at room temperature. Chromatin samples were sonicated for 10 cycles at 20 s ON/40 s OFF using a UR-20P (Tomy Seiko Co., Ltd. Tokyo, Japan) to produce DNA fragments of approximately 1,000 bp. Thereafter, aliquots of each sample were immunoprecipitated with pre-immune serum, anti-HAC1 antibody (PhytoAB, PHY7924A/Lot#20d58C6a), anti-acetyl-histone H3 antibody (EMD Millipore Corp. United States. 06-599/Lot#3927971), which recognizes histone H3 acetylated at the N-terminus, or anti-myc antibody (MBL International, 562/Lot#055) overnight at 4 °C. The chromatin–antibody complexes were precipitated using salmon sperm DNA/FG Protein G beads (Tamagawa Seiki Co., Ltd. Lot#345SNENCPG2) overnight at 4 °C. The ChIP samples were extracted with phenol/chloroform and precipitated using isopropanol. The amount of immunoprecipitated chromatin was determined using quantitative real-time PCR.

For Re-ChIP analysis, the 7-d-old *35S:4×myc-GAF1* transgenic seedlings were transferred to half-strength MS medium with or without 1 μM PAC or 10 μM GA_3_ and grown for 7 d. Chromatin was isolated from plants cross-linked with 1% (v/v) formaldehyde, and the first ChIP (IP1) was performed using pre-immune serum or an anti-myc antibody (MBL International, 562/Lot#055). Half of the sample was used to dissociate the chromatin–antibody complexes in re-ChIP buffer [50 mM Tris-HCl pH 8.0, 2 mM EDTA, 1% SDS, 10 mM DTT] at 37 °C. The second ChIP (IP2) was performed using pre-immune serum or an anti-HAC1 antibody (PhytoAB, PHY7924A/Lot#20d58C6a).

The primers used for ChIP-qPCR and Re-ChIP-qPCR analysis are listed in Table S2.

### EMSA

The EMSA experiments were performed as described previously (Fukazawa et al. 2014) with some modifications. pET30b:6×His-GAF1 was constructed as described previously (Fukazawa et al. 2014). Recombinant 6×His-GAF1 protein was expressed and affinity-purified from *E. coli* BL21 (DE3) pLysE using Ni^+^ resin (Novagen). The oligonucleotides were annealed and subsequently labeled using [α-^32^P] dCTP and the Klenow fragment of DNA polymerase I. The purified recombinant GAF1 protein was incubated with binding buffer (50 fmol labeled probe, 2 μg Poly dI/dC, 10% [v/v] glycerol, 20 mM Tris-HCl [pH 8.0], 3 mM MgCl_2_, 1 mM EDTA, 50 mM KCl, 2 μM ZnCl_2_) for 30 min on ice. DNA competitor was used at 500-fold excess molar concentration. The reactions were loaded by electrophoresis onto 3.6% polyacrylamide gels. The oligonucleotides used for EMSA are listed in Table S2.

### Transient expression in *Arabidopsis* mesophyll protoplasts

*ERF2* promoters with deletions from position −400 to +9 (+1, transcription start site) and −200 to +9 were cloned into the *Hind*III-*Bam*HI sites of the p-less LUC vector (Fukazawa et al. 2017). *ERF2* promoter was mutated to GAF1-binding sites using the megaprimer method (Barik 1996). *HAC1* promoters with deletion from position −1,900 to +9 (+1, transcription start site) were cloned into the *Hind*III-*Bam*HI sites of the p-less LUC vector (Fukazawa et al. 2017). The primers used for cloning are listed in Table S2. *Arabidopsis* mesophyll protoplasts were isolated using the Tape-*Arabidopsis* Sandwich method (Wu et al. 2009). The leaf epidermis of plants grown for 3 to 4 wks was removed and incubated in enzyme solution [1% cellulase, 0.25% macerozyme, 0.4 M mannitol, 10 mM CaCl_2_, 20 mM KCl, 0.1% BSA, 20 mM MES] with shaking for 1 h at room temperature. After washing with W5 solution [154 mM NaCl, 125 mM CaCl_2_, 5 mM KCl, 2 mM MES], the cells were resuspended in MMg solution [0.4 M mannitol, 15 mM MgCl_2_, 4 mM MES] followed by protoplasts preparation. The protoplasts, each plasmid, and PEG solution [40% (w/v) polyethylene glycol 4000, 0.2 M mannitol, 100 mM CaCl_2_] were mixed by pipetting and incubated at room temperature for 5 min. The cells were transferred to W5 solution and cultured under continuous light for 20 h at 22 °C. Luciferase activities were measured using a Dual-Luciferase Reporter assay System (Promega, Madison, WI, United States, Cat#E1960). The cell pellet was resuspended in Passive Lysis Buffer, and the cells were lysed by vortexing. After centrifugation at 15,500 g for 5 min at 4 °C, 10 μL of diluted sample was mixed with 50 μL of the Luciferase Assay Reagent Ⅱ in a 96-well plate and detected by a TriStar^2^ Multimode Reader LB 942 (Berthold Technologies, Bad Wildbad, Germany). Thereafter, 50 µL of Stop & Glo Reagent was added immediately, and the *Renilla* luciferase (rLUC) activity, serving as the internal standard, was measured. LUC activity was calculated by normalizing to rLUC activity. The data are presented as averages of 3 independent biological replicates.

### Statistical analysis

Two-tailed Student's *t*-tests were performed using Microsoft Excel. Significant differences were determined using one-way ANOVA followed by Tukey's post-hoc test in GraphPad Prism 10 software (GraphPad, Inc). Statistical significance was set at *P* < 0.05.

### Accession numbers

Arabidopsis Genome Initiative locus identifiers for the genes mentioned in this article are as follows: *HAC1* (At1g79000), *RGA* (At2g01570), *GAI* (At1g14920), *RGL1* (At1g66350), *RGL2* (At3g03450), *RGL3* (At5g17490), *GAF1/IDD2* (At3g50700), *CPS* (*GA1*) (At4g02780), *GA20ox2* (At5g51810), *GID1B* (At3g63010), *ELF3* (At2g25930), *ERF2* (At5g47220), *UBQ11* (At4g05050), and *ACT2* (At3g18780).

## Supplementary Material

koag235_Supplementary_Data

## Data Availability

The MS proteomics data have been deposited to the ProteomeXchange Consortium via the jPOST partner repository with the dataset identifier PXD079606.
